# Barriers to Care Leading to Fatal Consequences: A Case of Tuberculosis Sepsis Amid Policy Change

**DOI:** 10.7759/cureus.88918

**Published:** 2025-07-28

**Authors:** Zaineb Khawar, Mohana Chakkera, Danial Malik, Jihad Slim

**Affiliations:** 1 Internal Medicine, Saint Michael's Medical Center, Newark, USA; 2 Internal Medicine, St. Mary's General Hospital, Passaic, USA; 3 Infectious Diseases, Saint Michael's Medical Center, Newark, USA

**Keywords:** barriers to care, barriers to treatment, mycobacterium tuberculosis, political determinants of health, political factors, sepsis, septic shock, septic shock in disseminated tuberculosis, severe sepsis

## Abstract

*Mycobacterium tuberculosis* is an acid-fast bacterium with a diverse range of clinical manifestations and is considered rare in Western countries. Tuberculosis (TB) remains a global health concern and can pose several challenges in diagnosis and treatment. Paired with changing immigration policies, immigrant populations can face several barriers to healthcare. This case illustrates the impact of immigration policy on clinical outcomes and how government policies can act as a barrier to care. We present a case of a 26-year-old Guatemalan man with no known past medical history who came to the emergency department hemodynamically unstable with the presentation of abdominal perforation. He was urgently taken for an exploratory laparotomy, which revealed two perforations in the terminal ileum, extensive fibrinous exudate in the peritoneal cavity, and significant lymphadenopathy in the mesentery and retroperitoneum. The specimens collected intraoperatively were sent for evaluation and showed necrotizing granulomatous inflammation with transmural necrosis, and mesenteric lymph node biopsy revealed necrotizing granulomatous lymphadenitis, findings consistent with TB lymphadenitis. A sputum acid-fast bacilli (AFB) test further confirmed *Mycobacterium tuberculosis*. Given the concern for disseminated TB and ongoing ventilator and pressor support, this case required a multidisciplinary critical care course. The patient was started on antimycobacterial therapy with plans to transition to RIPE (rifampin, isoniazid, pyrazinamide, and ethambutol) therapy plus vitamin B6. The patients' hospital course was further complicated by loculated ascites with peritonitis and necrotizing pneumonia. Despite aggressive multidisciplinary medical therapy, the patient ultimately expired. The treatment of sepsis due to disseminated TB depends heavily on the ability to identify the causal infection, in addition to providing hemodynamic and organ support. The research stressing the timeliness of treatment and its impact on mortality in sepsis and miliary TB is well documented across medical literature. In this case, it is critical to analyze the political climate that may have prevented the patient from presenting to a healthcare provider when he first experienced symptoms.

## Introduction

*Mycobacterium tuberculosis* is an acid-fast bacterium with a predominant pulmonary manifestation and well-documented low prevalence in the United States and other Western countries [1]. However, tuberculosis (TB) remains a significant public health challenge in the US among migrant populations. In 2023, over 76% of reported TB cases occurred in non-US-born persons. The Centers for Disease Control and Prevention (CDC) reports that the highest risk of TB in the US is the origin of birth, with incidence rates in the migrant population nearly 19 times higher than the US-born population [2]. Despite increasing efforts to strengthen TB services in high-burden countries, the TB burden between US-born and non-US-born populations demonstrates this healthcare disparity [3].

Immigration status when non-US persons migrate to the United States drastically highlights further healthcare disparity, as this population is faced with unique barriers to medical care. Over the last decade, international migration has continued to rise despite the efforts of several countries to limit immigration opportunities. Research shows that undocumented immigrants underutilize the healthcare system, which not only puts their health at risk but, in the case of infectious disease, can be harmful to the population's health. Lack of insurance, fear of disclosure, and lack of social and financial support are the most common examples of obstacles the non-US population encounters [4].

In January 2025, there was a dramatic expansion of the US immigration enforcement policy. There was a drastic increase in US Immigration and Customs Enforcement (ICE) raids on immigrant communities, resulting in the arrest of over 65,000 migrants [5,6].

We describe a case of fatal sepsis secondary to disseminated TB in an undocumented Guatemalan patient who delayed seeking care during a time of drastic political unrest. This case illustrates the impact of immigration policy on clinical outcomes and how government policies can act as a barrier to care.

## Case presentation

In February 2025, a 26-year-old male from Guatemala, with no known past medical history, presented to the emergency department with worsening abdominal pain and poor appetite. On arrival, he presented with signs and symptoms of septic shock and was intubated for airway protection, given his hemodynamic instability. History was obtained from his brother, who reported the patient had experienced abdominal pain, fever, and nausea, but denied vomiting or hematemesis. The patient's family reported that the patient had been within the United States for over two years and denied a history of recent travel, sick contacts, prior surgeries, TB vaccination, trauma, medications, or allergies. The patient had a significant history of alcohol use, reportedly consuming 1-2 liters of hard liquor over three days, with his last intake occurring two to three weeks before symptom onset.

**Figure 1:**
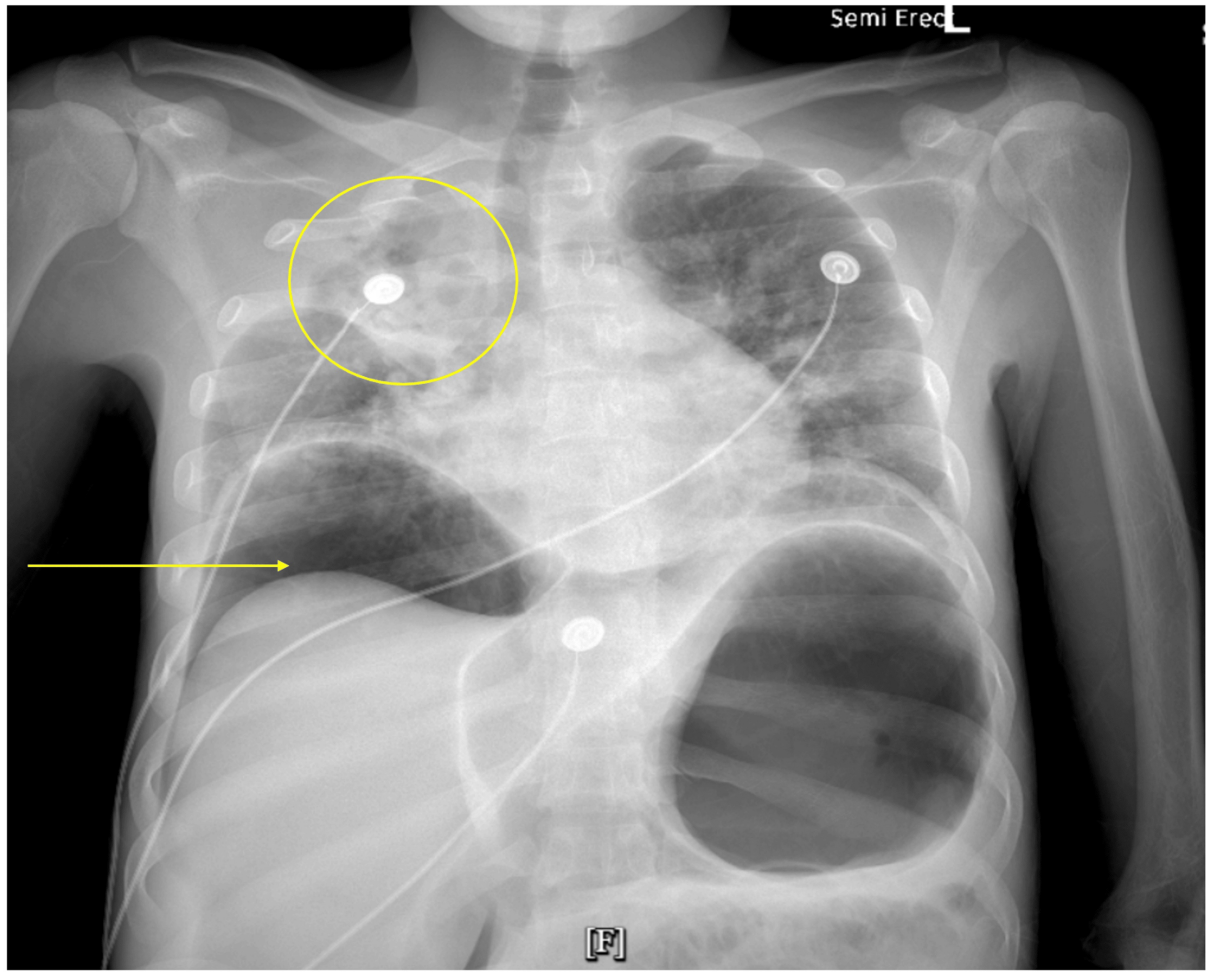
Chest X-ray on admission: free intraperitoneal air (arrow), gastric distention, and right upper lobe consolidation (circle).

Chest X-ray revealed a large amount of free intraperitoneal air, gastric distention, and right upper lobe consolidation (Figure 1). The patient was taken emergently to the operating room for exploratory laparotomy, which revealed two perforations in the terminal ileum, extensive fibrinous exudate in the peritoneal cavity, and significant lymphadenopathy in the mesentery and retroperitoneum. A right hemicolectomy and a few enlarged lymph nodes were resected, and specimens were sent for pathological evaluation.

Following exploratory laparotomy, the patient was received in the ICU on continued ventilation and sedation. On arrival to the ICU, the patient had persistent hemodynamic instability, indicating septic shock (Tables 1-3). The patient received aggressive fluid resuscitation and was initiated on two vasopressors, norepinephrine and vasopressin. Hypotension persisted, and the patient was started on stress-dose steroids (later tapered) and albumin. Due to extensive lymphadenopathy, the initial differential diagnosis included sepsis secondary to TB versus lymphoma. Empirical antibiotic therapy with piperacillin-tazobactam was initiated. The infectious diseases team was consulted, and recommended continuing piperacillin-tazobactam and adding a fluoroquinolone. Further diagnostic workup included HIV, *Histoplasma* antigen and serology, respiratory panel, mycobacterial cultures, *Mycobacterium tuberculosis* polymerase chain reaction (PCR), acid-fast bacilli (AFB) staining, and blood cultures. Pathological analysis of the ileum and lymph nodes biopsy taken from the operating room was pending to guide further management.

**Table 1:** Complete blood count on admission.

Laboratory test	Laboratory values	Reference Range
White blood cell	15.10 x 10^3^/uL	4.40-11.0 x 10^3^/uL
Hemoglobin	10.4 g/dL	13.5-17.5 g/dL
Hematocrit	32.6%	38.8-50%
Mean corpuscular volume	74.8 fL	81.2-95.1 fL
Platelets	451 x 10^3^/uL	150-450 x 10^3^/uL

**Table 2:** Complete metabolic panel on admission. CO2: carbon dioxide; BUN: blood urea nitrogen; AST: aspartate aminotransferase; ALT: alanine aminotransferase.

Laboratory test	Laboratory values	Reference range
Sodium	132 mmol/L	136-145 mmol/L
Potassium	5.0 mmol/L	3.5-5.3 mmol/L
Chloride	97 mmol/L	98-110 mmol/L
CO2	22.0 mmol/L	20-31.0 mmol/L
BUN	39.0 mg/dL	6.0-24 mg/dL
Creatinine	0.92 mg/dL	0.70-1.30 mg/dL
Glomerular filtrate rate	>90.0 mL/min/1.73m^2^	>90.0 mL/min/1.73m2
Calcium	8.3 mg/dL	8.6-10.4 mg/dL
AST	63 U/L	10-36 U/L
ALT	24 U/L	10-49 U/L
Total protein	6.4 g/dL	6.4-8.4 g/dL
Total bilirubin	1.3 mg/dL	0.2-1.2 mg/dL
Albumin	3.2 g/dL	3.2-4.8 g/dL
Glucose	148 mg/dL	70-140 mg/ dL
Anion gap	13.0 mmol/L	6-19 mmol/L

**Table 3:** Inflammatory markers on admission.

Laboratory test	Laboratory value	Reference range
C-reactive protein	32.2 mg/dL	0.0-0.8 mg/dL
Procalcitonin	18.73 ng/mL	0.0-0.5 ng/mL
Lactic acid	3.02 mmol/L	0-2 mmol/L

Initial pathology suggested TB, and sputum AFB testing confirmed *Mycobacterium tuberculosis*. Given the concern for disseminated TB and ongoing ventilator and pressor support, infectious disease specialists initiated IV antimycobacterial therapy: rifampin, amikacin, linezolid, ciprofloxacin, and metronidazole for post-peritonitis anaerobic coverage, with plans to transition to RIPE (rifampin, isoniazid, pyrazinamide, and ethambutol) therapy plus vitamin B6 once enteral intake was possible. Based on drug levels, amikacin was increased to 15 mg/kg/day, and ciprofloxacin was replaced with moxifloxacin. Tissue cultures also grew *Klebsiella pneumoniae*,* Enterococcus faecium*, and coagulase-negative *Staphylococcus*. *Cryptococcal* antigen, *Histoplasma* antigen antibody, and HIV testing were negative. Pathology of the terminal ileum showed necrotizing granulomatous inflammation with transmural necrosis, and mesenteric lymph node biopsy revealed necrotizing granulomatous lymphadenitis, findings consistent with TB lymphadenitis. So, intravenous antimycobacterial treatment was continued.

One week into admission, the patient developed a fever. Common causes, including line-related infections, were ruled out. Repeat blood cultures were obtained, and the same antimicrobial therapy was continued. He developed hypernatremia with high urine output, likely due to diabetes insipidus related to TB or medications. Nephrology was consulted, and sodium was normalized after one dose of desmopressin and dextrose. The patients' clinical course was further complicated by abdominal distension, and CT of the abdomen revealed loculated ascites with peritonitis and necrotic abdominal and inguinal lymphadenopathy. The persistent fever and leukocytosis were suspected to be due to superimposed bacterial peritonitis. Interventional radiology drained 500 cc of exudative fluid; cultures were pending. As per surgical recommendations, enteral feeding was initiated via nasogastric tube, and intravenous antimicrobial therapy was transitioned to RIPE.

Despite negative repeat blood cultures, a new pulmonary infiltrate on chest X-ray prompted initiation of piperacillin-tazobactam for possible secondary lung infection in addition to RIPE. Ascitic fluid later grew pan-sensitive *Escherichia coli*, confirming bacterial peritonitis, and piperacillin-tazobactam was continued. A repeat CT of the abdomen showed moderate loculated ascites, which was drained again. Due to a prolonged ICU stay and broad-spectrum antimicrobial exposure, the patient was started on high-dose extended meropenem (2 g every eight hours). With continued fever and negative repeat peritoneal cultures, meropenem was later replaced with moxifloxacin and metronidazole alongside RIPE. Serial AFB smears showed a declining bacillary load, from AFB +4 on admission to +1 throughout treatment, indicating a response to therapy.

The patient was weaned off vasopressors but remained dependent on mechanical ventilation. Due to the anticipated prolonged need for ventilatory support, a tracheostomy was performed. Bronchoscopy revealed normal airway anatomy with minimal secretions; bronchial cultures, Fungitell, and cytology were negative. Gastroenterology was consulted for percutaneous endoscopic gastrostomy (PEG) placement, but it was deferred pending the resolution of peritonitis and ascites. Enteral nutrition was continued via the nasogastric (NG)/orogastric (OG) tube.

**Figure 2:**
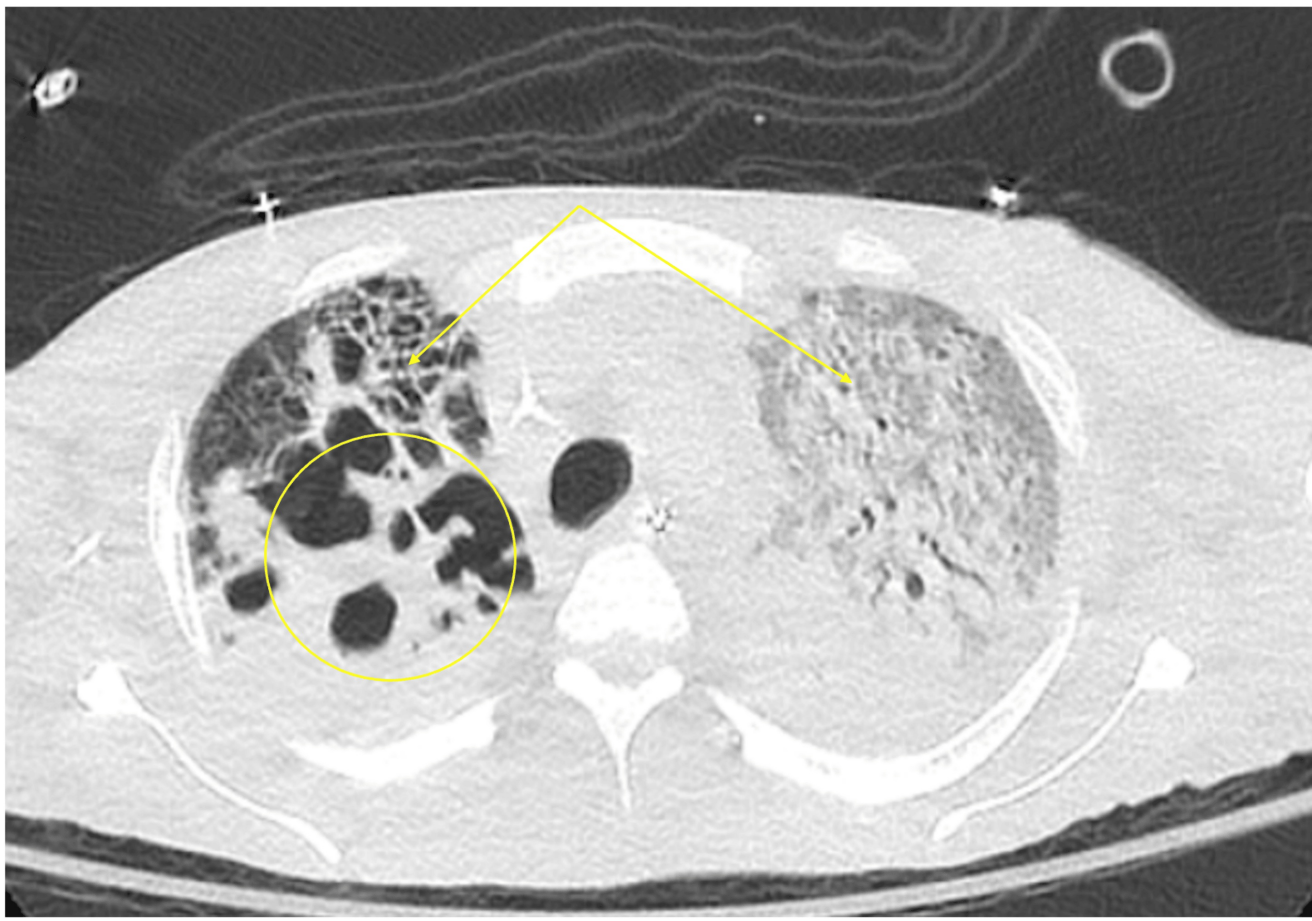
CT of the chest with severe bilateral necrotizing pneumonia (arrow) with cavitations in the right upper lobe (circle).

The patient continued to have low-grade fevers and worsening pulmonary infiltrates. A CT of the chest revealed severe bilateral necrotizing pneumonia with cavitations in the right upper lobe (Figure 2). With gram-negative rods isolated from sputum, meropenem was restarted, and metronidazole was discontinued. The patient later became afebrile; sputum culture grew *Enterobacter* that was sensitive to fluoroquinolones, so meropenem was discontinued and moxifloxacin was continued. Despite initial stabilization, the patient experienced cardiac arrest and achieved return of spontaneous circulation (ROSC) after four cycles of CPR. However, he suffered a second cardiac arrest within an hour and subsequently expired.

## Discussion

Our case demonstrated the fatal presentation of disseminated TB in a 26-year-old undocumented Guatemalan man who presented with bowel perforation, which was found to be a consequence of miliary TB. Sepsis and septic shock continue to be important contributors to morbidity and mortality worldwide; however, TB remains a rarely reported cause of septic shock. TB infects nearly one-third of the world’s population, resulting in approximately 1.7 million deaths each year. Although the incidence of TB in the US is low, the proportion of infected non-US-born patients continues to remain relevant both clinically and concerning public health [7]. Disseminated TB represents a severe form of extrapulmonary infection involving hematogenous spread to multiple organ systems and is associated with high mortality, particularly as diagnosis can be delayed due to its chronic etiology [8]. Research analyzing over 50 cases of sepsis due to TB shows that the prognosis for TB septic shock is abysmal, and data suggest that delayed initiation of appropriate antimicrobial therapy and delay in medical treatment due to failure to recognize the causal disease contribute significantly to the high mortality rates [7]. In another systematic analysis analyzing over 20 cases of TB sepsis in non-HIV patients, it was found that 64% of patients died within 30 days of presentation, and that the average time lapse from presentation to starting the empiric anti-tuberculous treatment regimen was six days. This analysis also confirmed that mortality was related to delays in starting the anti-TB treatment regimen [9].

TB primarily presents as a pulmonary disease; however, over a third of cases involve other organ systems. TB pathophysiology involves contamination via air droplets and infiltration of the lung epithelial macrophages. The rampant bacterial proliferation eventually reaches the lymphatic ducts and, without a sufficient T-cell response, leaves the body susceptible to systemic spread. Abdominal TB, as seen in this case, typically occurs due to hematogenous spread from a pulmonary focus or by local gastrointestinal spread. Intestinal TB usually presents with fever, nutrient deficiencies, and possible intestinal obstruction, which may lead to bowel perforation [10]. The diagnosis of miliary TB includes history and physical examination and is supported by laboratory and radiologic findings. Patients who present in the US or other Western countries are likely to be migrants from a TB endemic region, most commonly within the first year of migration [2]. Patients will complain of recurrent fevers, weight loss, or chronic night sweats. Laboratory findings may include hematologic abnormalities, including but not limited to pancytopenia, anemia, or leukocytosis with predominant lymphocytosis. They may also present with elevated inflammatory markers, and biochemistry panels may be normal or have subtle electrolyte disturbances. Typically, renal involvement is not appreciated. A chest radiograph will support the diagnosis of TB and show cavitary lesions or opacities in the upper lobes [11]. Miliary TB chest radiography will show homogeneously distributed, uniform millet-shaped lesions, known as miliary mottling, in the lung apices [10]. Disseminated TB can be further assessed by computed tomography or ultrasonography. For definitive diagnosis, sputum, body fluid, or biopsy samples will show granulomatous inflammation with central caseation with or without tubercle bacilli. Blood cultures are usually not required for diagnosis, but may be positive in miliary TB cases [10].

The treatment of sepsis due to disseminated TB depends heavily on the ability to identify the causal infection, in addition to providing hemodynamic and organ support [7]. Our patient was monitored in the intensive care unit as a postoperative case and was maintained on mechanical ventilation. Standard treatment regimen for TB consists of a two-month induction phase with isoniazid, rifampicin, pyrazinamide, and ethambutol, followed by a four-month continuation phase with isoniazid and rifampicin [10]. This combined six-month therapy is also an effective treatment regimen for patients with abdominal TB [12]. Delayed treatment can lead to several complications in the disease course, including but not limited to acute respiratory distress syndrome, tubercular empyema, pneumothorax, endocarditis, disseminated intravascular coagulation, and bone marrow suppression. Postponement of adequate treatment is the most significant factor responsible for mortality. The mortality related to miliary TB is upwards of 30% in adults [10].

The research stressing the timeliness of treatment and its impact on mortality in sepsis and miliary TB is well documented across medical literature. In this case, it is critical to analyze the political climate that may have prevented the patient from presenting to a healthcare provider when he first experienced symptoms. In addition to barriers to care like insurance, language challenges, distrust in the medical system, or poor healthcare literacy, rapid changes made to immigration policy coinciding with his illness resulted in delayed presentation to healthcare [4]. Our patient's undocumented status and delayed presentation raise important ethical and clinical concerns. The January 2025 ICE raids were widely publicized and showed aggressive practices in public spaces and workplaces. This understandably generated fear in local immigrant communities, even in cities or locations considered “sanctuaries” [13].

## Conclusions

This case represents a complicated clinical course surrounding disseminated TB infection with abdominal perforation and septic shock at a time of political unrest. From discussions with the patient’s family, it became evident that the patient experienced symptoms for several weeks before presenting to the hospital, but avoided seeking care due to the fear of immigration enforcement. This case displays how hesitancy to receive medical care due to fear is a barrier to healthcare and a factor that can alter a patient's clinical course.

As stated previously, the treatment of sepsis due to disseminated TB depends heavily on the ability to identify the causal infection, in addition to providing hemodynamic and organ support promptly. Beyond the clinical understanding of TB, this case illustrates unique and policy-driven barriers to care. In this case, we must consider that our patients' delay in seeking care due to fear of deportation contributed to the progression of TB, an otherwise treatable pulmonary infection, to develop into disseminated disease, resulting in abdominal perforation, septic shock, and eventually death. As clinicians, we must recognize the broader determinants of health and approach our patients, particularly those part of the immigrant community, with these factors in mind.
